# Measurement Invariance and Differential Item Functioning Across Gender Within a Latent Class Analysis Framework: Evidence From a High-Stakes Test for University Admission in Saudi Arabia

**DOI:** 10.3389/fpsyg.2020.00622

**Published:** 2020-04-03

**Authors:** Ioannis Tsaousis, Georgios D. Sideridis, Hanan M. AlGhamdi

**Affiliations:** ^1^Department of Psychology, University of Crete, Rethymno, Greece; ^2^Boston Children’s Hospital, Harvard Medical School, Boston, MA, United States; ^3^National and Kapodistrian University of Athens, Athens, Greece; ^4^National Center for Assessment in Higher Education, Riyadh, Saudi Arabia

**Keywords:** latent class analysis, Differential Item Functioning, mixture modeling, auxiliary variables, high-stakes testing, multiple indicator multiple causes

## Abstract

The main aim of the present study was to investigate the presence of Differential Item Functioning (DIF) using a latent class (LC) analysis approach. Particularly, we examined potential sources of DIF in relation to gender. Data came from 6,265 Saudi Arabia students, who completed a high-stakes standardized admission test for university entrance. The results from a Latent Class Analysis (LCA) revealed a three-class solution (i.e., high, average, and low scorers). Then, to better understand the nature of the emerging classes and the characteristics of the people who comprise them, we applied a new stepwise approach, using the Multiple Indicator Multiple Causes (MIMIC) model. The model identified both uniform and non-uniform DIF effects for several items across all scales of the test, although, for the majority of them, the DIF effect sizes were negligible. Findings from this study have important implications for both measurement quality and interpretation of the results. Particularly, results showed that gender is a potential source of DIF for latent class indicators; thus, it is important to include those direct effects in the latent class regression model, to obtain unbiased estimates not only for the measurement parameters but also of the structural parameters. Ignoring these effects might lead to misspecification of the latent classes in terms of both the size and the characteristics of each class, which in turn, could lead to misinterpretations of the obtained latent class results. Implications of the results for practice are discussed.

## Introduction

Standardized testing for university admission has seen enormous growth over the last decades and affects the lives of millions of young individuals around the globe. Several different educational achievement tests are used as criteria for university entrance, and students obtain access to higher education level based on their performance on these tests (House, 1997). Most admissions tests are composed of different thematic areas, such as verbal, numerical, and analytical reasoning skills or discipline-specific knowledge (e.g., Mathematics, Physics, etc.) since it has been found that the combination of several of these skills is a necessary condition for a successful degree completion in all fields of study (Kuncel and Hezlett, 2007). In a recent study, Noble and Camara (2003) reported that more than 80% of all 4-year universities and institutions in the U.S. require an admissions test, and more than 91% of non-open institutions required one. Moreover, more than 1.5 million students take admissions tests in the U.S. per year, and this number is constantly increasing. Along the same lines, in Saudi Arabia, the Grade Point Average (GPA), along with other standardized test results, are the requirements for admission to higher education institutions. These tests are the *Standard Achievement Admission Test* (SAAT) which has been developed to measure domain-related knowledge (e.g., math, physics, biology, chemistry), and the General Aptitude Test (GAT) that focus more on the students’ capability for learning. The composite scores (GPA, SAAT, GAT) are usually employed to inform the decision-making process.

One important reason why these tests have gained such popularity is that they offer objectivity over other available criteria, such as high school grades, structured interviews, etc. (Phelps, 2005; Benbassat and Baumal, 2007). Another advantage is, that these tests are norm-referenced, in that the individual’s scores are compared with the scores of a group of people (i.e., normative sample), who share the same characteristics and attributes such as gender, age, socio-economic status, etc. Furthermore, a standardized test has certain rules in terms of administration, since all test takers complete it following the same directions and time restrictions. Additionally, the evaluation of the student’s performance is not affected by subjective factors (e.g., evaluator’s perceptions), since the scoring of the test is based on a series of pre-set, objective, criteria. Finally, the interpretation of the obtained scores is also free of subjective inferences and context (e.g., class size, teacher’s quality, etc.), but rather on the test-takers’ relative information about their readiness to undertake university coursework.

A serious issue in educational and psychological testing, with lasting and at times, serious consequences in test-takers’ present and future life, is fairness (Sackett et al., 2008). According to Educational Testing Service (2019), fairness “…is the extent to which the inferences made on the basis of test scores are valid for different groups of test-takers” (p.19). Especially for standardized high-stakes tests, fairness is a crucial issue, since these instruments serve as a gateway for admitting individuals into higher education (Caines et al., 2013). From a more technical perspective, the concept of fairness in testing is equivalent to bias. Test or item bias is related to all those construct-irrelevant factors such as gender, age, race, culture, socio-economic status, education, etc., which can result in systematic distortion on the performance of test-takers from different groups, although they possess the same level of the underlined ability or trait (Millsap and Everson, 1993).

With regard to gender, there is a long-lasting tradition pointing to the superiority of males in STEM-related subjects (e.g., math, engineering) over females (Wai et al., 2010; Lakin, 2013) but these findings are far from being conclusive as girls appear to have higher school grades in STEM subjects compared to males (Voyer and Voyer, 2014). Interestingly, and regardless of the empirical evidence, males dominate these fields professionally, and potential explanations put forth include (a) females conform to stereotype threat (Spencer et al., 2016), and (b) the variability hypothesis (Johnson et al., 2008). With regard to the stereotype threat, females tend to behave for rather than against gender stereotypes being fearful of the risk of backlash (Rudman and Phelan, 2008). The variability hypothesis, on the other hand, suggests that the observed higher variability in achievement in males (Reinhold and Engqvist, 2013) suggests an excess of males on the top 10% of the achievement’s distribution (Halpern, 2007) although that is not always the case (Wang and Degol, 2017). Perhaps the most salient evidence regarding gender stereotypes in math and STEM subjects comes from a recent meta-analysis by O’Dea et al. (2018) who analyzed data involving 1.6 million students. The overarching conclusion from this analysis was that the variability hypothesis did not hold as equal numbers of males and females belonging to the top 10% of their class in stem-related subjects. Consequently, the null hypothesis of no differences in math across gender seems to currently prevail using all available evidence. If this finding holds, then it becomes even more important to evaluate the existence of potentially biased items in measures of math achievement across gender, which is the primary purpose of the present study.

To date, efforts to examine item and test bias are coming from the two basic traditions in measurement theory: (a) from the perspectives of the Classical Test Theory (CTT), the Multiple-Group CFA (MGC-FA) approach, in which the relations between observed variables and latent construct(s) are tested for measurement invariance between groups (Vandenberg and Lance, 2000), and, (b) from the perspectives of modern methods such as Item Response Theory (IRT), and the Differential Item Functioning (DIF) approach, in which item behavior is evaluated on whether it assesses equivalent levels on the latent trait across members of separate groups (Roussos and Stout, 1996; Embretson and Reise, 2000). A more recent approach is the Multiple Indicators and Multiple Causes (MIMIC) modeling procedure, in which a covariate exerts direct effects on both, the latent variable(s), and the factor indicators (Finch, 2005). The MIMIC model is considered as a special form of Structural Equation Modeling (SEM) since it integrates causal variables (i.e., covariates) within a confirmatory factor analysis model (MacIntosh and Hashim, 2003). When a MIMIC model, is utilized, two different models are tested: a *measurement model*, in which the relationship between a latent variable and its indicators is tested (i.e., items) and a *structural model*, in which the direct effect of a covariate that defines group membership (e.g., gender) on factor means and factor indicators (items) are also tested. A significant direct effect of the covariate on the latent factor indicates that factor means are different at the different levels of the covariate (e.g., males vs. females). Similarly, a significant direct effect of the covariate on an item of the scale suggests that the item mean is different at the different levels of the covariate, after controlling for the latent factor mean.

Raykov et al. (2013), argued that MIMIC models are advantageous in examining DIF over other analogous techniques such as multi-group CFA (Vandenberg and Lance, 2000; Millsap, 2011). First, MIMIC models require smaller sample sizes as by modeling one group (over two groups), the number of estimated parameters is dramatically decreased, and thus, the size of the input matrix is less demanding (Brown, 2015). Another advantage of this model is that it simultaneously addresses four issues: (a) estimation of IRT measurement parameters, which provides evidence for the internal validity of the measure; (b) examination of the relationship between the covariate and the latent construct, which provides information for the external validity of the measure; (c) examination of Differential Item Functioning (DIF) across the different levels of the covariate, and, (d) relaxation of the assumptions of unidimensionality and local dependency, which are very important in CFA and IRT models. Finally, MIMIC modeling allows for the simultaneous evaluation of the effect of the covariates on both, the latent variable(s) as well as the factor indicators, and all obtained estimates are adjusted for all other covariates in the model (Muthén et al., 1991).

The MIMIC approach is mainly used by applied researchers within the factor analytic framework (Finch, 2005; Willse and Goodman, 2008; Woods and Grimm, 2011). However, this approach can easily be extended to a Latent Class (mixture modeling) framework, especially when researchers are interested in understanding the nature of the emerging classes and the characteristics of the people who comprise them (Nylund-Gibson and Choi, 2018). For example, there are cases where researchers are interested in examining the effect of covariates or distal outcomes on latent class membership. Particularly, with this approach, we can examine whether there are direct effects from covariates to latent variable indicators, in an attempt to identify possible sources of DIF across the covariate’s levels (Masyn, 2017), or whether the obtained latent-classes display statistically significant mean-level differences in an outcome variable (Nylund-Gibson and Choi, 2018). Moreover, we can examine whether the obtained latent classes are *invariant* across two or more groups, with every individual within a class having the same expected response on each item (Kankaraš et al., 2011).

An issue that needs attention when examining the effect of a covariate (or a distal variable) on the latent class variable is, how we examine these two components: do we examine their relationship within a 1-step procedure (i.e., a simultaneous estimation of the latent class measurement model and the association between the latent class and the covariate) or we examine them via a stepwise procedure (i.e., first we estimate the latent classes without the covariate, and then we examine the association between the latent class variable and the covariate). Previous studies have shown that the first approach, although sensible (this is how it works in SEM), it could lead to the distortion of the latent class results in terms of the estimation of the latent class probabilities and the conditional probabilities. This, in turn, might lead to misspecification of the latent classes in terms of both the size and the characteristics of each class (Vermunt, 2010; Asparouhov and Muthén, 2014; Nylund-Gibson et al., 2014).

Today, there is general agreement among experts that the best method to overcome this problem is first to determine the number of latent classes needed to describe the population homogeneity adequately without the presence of the covariate. The second step is to examine the association between the covariate and the latent class variable (Muthén et al., 1991; Vermunt, 2010; Nylund-Gibson and Masyn, 2016). Among the different suggested approaches (more than seven are reported in the literature), the most advanced is the three-step procedure. The measurement parameters of the latent classes are held fixed (step 1) while accounting for classification error (step 2), and then the covariate is introduced in the model, and its relationship to the latent class variable is estimated (step 3) (see Nylund-Gibson et al., 2014 for a detailed description). However, even though this approach has been recently criticized for not being robust to avoid misspecifications in the latent class enumeration process (i.e., determining the optimal number of classes) and obtain accurate estimates of the covariates’ effects on latent class membership (Kim et al., 2016; Masyn, 2017).

The basic idea in measuring invariance or non-invariance (DIF) in a latent class model is simple: first, a model in which all response probabilities are allowed to vary across groups is examined; then, a model where these probabilities are constrained to be equal is examined, and the two models are compared using a difference likelihood ratio test. If the constrained model is significantly worse compared to the unconstrained model, invariance across classes is lacking. At the last stage, individual indicators are examined for the presence of uniform and non-uniform DIF. The difference between the two types of DIF exists in the shape of the item response functions. Uniform DIF exists when the item response pattern is related to the group at all levels of the latent class variable and does not depend on the latent ability level, whereas in non-uniform DIF, the IRFs across groups cross.

On the other hand, non-uniform DIF exists when the differences depend on the latent class levels (Berger and Tutz, 2016). Recently, measurement invariance within the latent class paradigm has been examined either by use of the multi-group latent class analysis (MGLCA) (for an extended review and detailed description, see Collins and Lanza, 2010) or the MIMIC approach. In previous years, some studies have used MGLCA to examine whether the nature of latent classes differs across known subgroups in the populations (e.g., Lohman et al., 2014; Finch, 2015). The MIMIC approach to test for measurement invariance, however, is new, and since its introduction (Masyn, 2017), there is to our knowledge no other study that utilized the method using real data. Consequently, in the present study, we follow the stepwise protocol presented by Masyn (2017), to examine potential sources of DIF across gender using a high-stake admission test for university entrance (i.e., Standard Achievement Admission Test - SAAT).

## Materials and Methods

### Participants and Procedure

The sample used in this study consisted of 6,260 participants. From them, 3,563 (56.9%) were males, and 2,697 (43.0%) were females. Five participants (0.1%) did not report their gender and were excluded from the study. Concerning the region of residence, participants came from all 13 provinces of the Kingdom of Saudi Arabia, with the majority coming from three urban areas: 1,469 (23.4%) from Riyadh, 1,246 (19.9%) from Makkah, and 513 (13.3%) from Eastern Province. No missing data for this variable were reported. T he study was conducted as part of a National Examination in Saudi Arabia and was reviewed and approved by the National Center for Assessment in Higher Education (Qiyas) Ethics Committee^1^. All participants were informed that their responses would be utilized as a part of a larger study to evaluate the psychometric properties of the measure. Completion of the test comprised their informed consent for their participation. No participants reported any psychological or emotional issues that would inhibit their full performance.

### Measure

#### Standard Achievement Admission Test (SAAT; National Center for Assessment, 2012)

The SAAT is a high-stakes standardized test used for university admissions in the Kingdom of Saudi Arabia. The test was designed to assess students’ readiness for higher education and covers four basic academic domains (subject areas): Mathematics, Biology, Chemistry, and Physics, and focuses on the material of the official 3-year (scientific) curriculum of the Saudi Arabia High Schools. For this study, a shortened version of the SAAT comprised of 65 multiple-choice items (4 alternative options) was used. The content of the items was distributed as follows: 20% of each subject for the first year of the high school syllabus, 30% of each subject for the second year of the high school syllabus, and 50% of each subject for the third year of the high school syllabus. The test time for each section is 25 min. The total time of the test, including the time given to instructions, is 2 h. The SAAT has been developed and evaluated within the IRT framework and exhibits excellent psychometric characteristics. Due to space constraints, only the Math scale was analyzed. However, all analyses reported in this study were also conducted for the remaining scales, and they are available upon request. The Math scale constitutes of a unidimensional construct (CFI = 0.964, TLI = 0.958, RMSEA = 0.019, and SRMR = 0.024). The mean difficulty level of the test was 0.45 (0.33 for the Math scale), while the mean item discrimination was 0.30 (0.24 for the Math scale) (Luo and Al-Harbi, 2016). The SAAT also exhibits acceptable internal consistency levels (total omega = 0.88), although, for the Math scale, the omega index was somewhat lower (i.e., 0.60).

### Analytical Strategy

First, a Latent Class Analysis (LCA, Lazarsfeld and Henry, 1968; Goodman, 1974) was conducted to identify the optimal number of distinct groups (i.e., classes) that meaningfully differentiate item responses among participants. The LCA model, utilizes two types of parameters (the proportion of the population belonging to a particular class, and the probability of an individual to answer an item correctly given that he/she has been classified in a particular class), to identify subgroups of participants who share similar patterns of responses. A typical statistical approach in LCA is to examine the fit of several models with different numbers of latent classes, and then to compare them using different inferential and information criteria of model fit.

Model fit of an estimated LCA model is usually tested via the likelihood-ratio (L^2^) chi-square test. However, the most interesting process in LCA is not the evaluation of the fit of a model, but rather which of the contrasted models fits the data better. In this case, it is not valid to subtract the Ls^2^ and the corresponding df values of the compared models and test for significance, because this conditional test does not have an asymptotic chi-squared distribution (Vermunt and Magidson, 2004). Instead, the Vuong-Lo-Mendell-Rubin (LMR) chi-square test (i.e., two times the loglikelihood difference value for the respective different number in estimated parameters) seems more appropriate to determine the best fitting model among the contrasted nested latent class models (Vuong, 1989; Lo et al., 2001).

On the other hand, it is well known that chi-square goodness-of-fit tests are sensitive to large samples since they tend to mistakenly reject the null hypothesis (Agresti, 2013) even when deviations from a perfect model are negligible. For that, several alternative statistical criteria have been suggested for deciding the optimum model solution in LCA (Masyn, 2013), although, as Muthén and Asparouhov (2006) pointed out, there is no single method for comparing models with different numbers of latent classes that is widely accepted. Thus, in this study, we decided to employ several different information criteria to decide on the optimal model solution. First, we used the Akaike’s (AIC) and Bayesian Information Criteria (BIC) (also termed penalized statistics), as well as some of their variants to control for excessive power due to the large sample size (>6,000 participants). Recent simulation studies have shown that the BIC performs better than other information criteria and likelihood ratio tests in identifying the appropriate number of latent classes (Nylund-Gibson et al., 2007). Additionally, we adopted the BIC’s sample size correction variant, the consistent AIC (CAIC; Bozdogan, 1987), the Approximate Weight of Evidence criterion (AWE; Banfield and Raftery, 1993), and the Schwartz Information Criterion (*SIC*; Schwarz, 1978). Moreover, additional quantitative indices were employed such as the approximate Bayes Factor (BF), which tests the relative fit between two competing models, the approximate correct model probability index (cmP), which compares all models with the sum value being 1, assuming one of the tested models is the correct model (Masyn, 2013). The LCA was performed using Mplus, version 8.3 (Muthén and Muthén, 1998-2019), using the Robust Maximum-likelihood (MLR) as a method of estimation and the expectation-maximization (EM) algorithm.

### Stepwise Procedure for Testing for DIF

**FIGURE 1:**
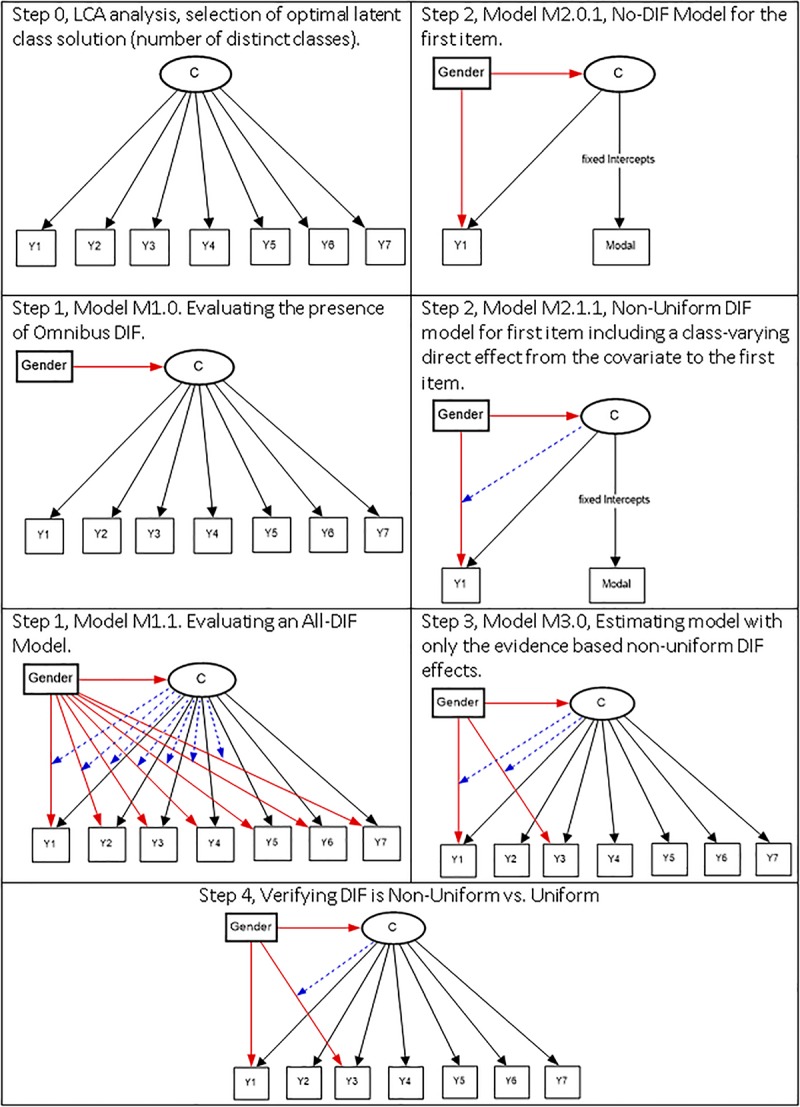
Stepwise procedure for DIF detection using mixture modeling.

The following stepwise procedure has been developed by Masyn (2017), and we draw heavily from the original source. Graphically the steps are shown in Figure 1. The interested reader may also consult (Masyn, 2013).

#### Step 0, Selecting the Optimal Latent Class Solution

This first step involves the series of latent class models that are run to satisfy and conclude the latent class enumeration process and select the optimal model. Note that during this process, the covariate is treated as an auxiliary variable not to have any influence on the latent class solution formation.

#### Step 1, Evaluating Omnibus DIF

This step involves comparing two nested models. The first, termed M1.0, includes the regression of the covariate on the latent class variable only, not the indicators (Omnibus test), which evaluate the appropriateness of a no-DIF model. This model is contrasted to a model in which the latent variable and the indicators are regressed onto the covariate (M1.1), which is an all-DIF model as the effects of the covariate on the items are left free to vary across classes (non-uniform DIF). In the presence of DIF, a comparison of the two models by use of a likelihood ratio test should provide evidence in favor of model M1.1 compared to M1.0. If Model M1.0 is the preferred model, then there is no evidence of significant DIF due to the covariate. Preference for model M1.1., however, requires further scrutiny over the location of non-invariance due to the covariate.

#### Step 2, Evaluating the Presence of Non-uniform DIF

Assuming preference for lack of measurement invariance model (i.e., M1.1), a series of models are run to evaluate the effects of the covariate on each one of the indicators to test the hypothesis that non-uniform DIF is present. This step involves first saving the model assignment from a previous run, defining it as a nominal variable, and then using the class-specific multinomial intercepts of the modal assignment (reflecting classification error rates) as indicators in a new LCA model. The compared models include a no-DIF model (termed M2.01) in which the latent class c is regressed onto the covariate (e.g., gender) and a, one-item at a time, DIF model in which an item is regressed onto the covariate (termed M2.1.1 for the first item, M2.1.2 for the second item, etc.). Models’ comparison will involve a series of likelihood ratio difference tests. Evidence in favor of the later model will signal the presence of non-uniform DIF for that item in the given run.

#### Step 3, Selecting Most Parsimonious Non-uniform DIF Model

The goal of this step is to identify the model in which only significant DIF effects are included. Consequently, a new model termed M3.0 involves estimating a latent class model with all non-uniform paths (from step 2) that were identified as being statistically significant, and we term this model as the “Parsimonious Non-Uniform DIF model.” This model (M3.0) should then be contrasted with two comparison models. The first comparison involves contrasting the M.3.0 model to the no-DIF model (M1.0) in step 1 with the expectation that M3.0, the parsimonious non-uniform DIF model, would be superior to model M1.0, the no-DIF model. The second comparison involves contrasting M3.0, the parsimonious non-uniform DIF model with M1.1, the all DIF model. The expectation here is that the parsimonious non-uniform DIF model (i.e., M3.0) would be no worse than the all DIF model.

#### Step 4, Testing for Uniform DIF

The goal of this step is to test the hypothesis that the earlier identified as exerting non-uniform DIF effects items were not exerting uniform effects. Consequently, the model in step 3 in which non-uniform DIF effects were identified (M3.0) was contrasted with models in which the effects of the covariate on each item earlier identified as exerting varying influences across classes was consistent across classes (i.e., uniform). Evidence of non-uniform DIF effects would be manifested with non-significant differences between models M3.0 and M4.1–M4.7 in that there is insufficient evidence that the effect of the covariate on the item is non-uniform (because models with uniform and non-uniform effects are no different). A significant difference, favoring the M4 models would suggest that fixing the effects of the covariate on an item to be invariant across classes (i.e., uniform DIF) is preferred. All DIF analyses were performed using Mplus, version 8.3 (Muthén and Muthén, 1998-2019). The syntax code can be found in the Supplementary Material that accompanies this manuscript.

### Differential Item Functioning: Effect Size Conventions

Several studies have looked upon the issue of effect size metrics for DIF (e.g., Raju, 1990; Zwick, 2012) suggesting diverse analytical means (e.g., Raju, 1990; Raju et al., 1995; Penfield and Lam, 2000 etc.). Amongst them, the most prominent are the ETS criteria, which transform the difference logit parameter onto the delta metric system (Holland and Thayer, 1988; Linacre and Wright, 1989; Dorans and Holland, 1993). Conventions on effect sizes, based on the difference logit parameter, are 0.44 and below pointing to the existence of negligible DIF, and estimates greater than 0.64 showing large DIF. Lin and Lin (2014) extended the ETS criteria to avoid Type-I errors with values of 0.45 and below being indicative of negligible DIF, values between 0.45 and 0.90 in logits representing medium level DIF, and values greater than 0.90 showing large DIF. The present study includes all conventions.

## Results

### Class Enumeration Process

Step 0 in the evaluation of DIF goal involves the prerequisite selection of an optimal latent class model. The class enumeration process involves contrasting adjacent latent class models (from 1 to, e.g., 4 or 5 classes) by use of likelihood ratio difference tests and information criteria. Table 1 presents the results from this process for the Mathematics scale.

**TABLE 1:** Latent class enumeration process of SAAT mathematics sub-scale (*N* = 6265).

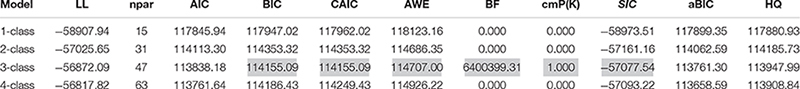

*LL, Model maximum log-likelihood value; npar, number of parameters; AIC, Akaike’s information criterion; BIC, Bayesian information criterion; CAIC, consistent Akaike’s information criterion; AWE, average weight of evidence criterion; BF, approximate Bayes factor comparing row model (K classes) to model with one additional latent class (K + 1 classes) in the row below; cmP(K) = approximate correct model probability for the row model (K classes) compared to all other models in the table; *SIC*, Schwartz Information Criterion; aBIC, adjusted sample size BIC; HQ, Hannan and Quinn information criterion. Shaded cells show preferred solution based on diverse criteria for the LC enumeration process.*

**FIGURE 2:**
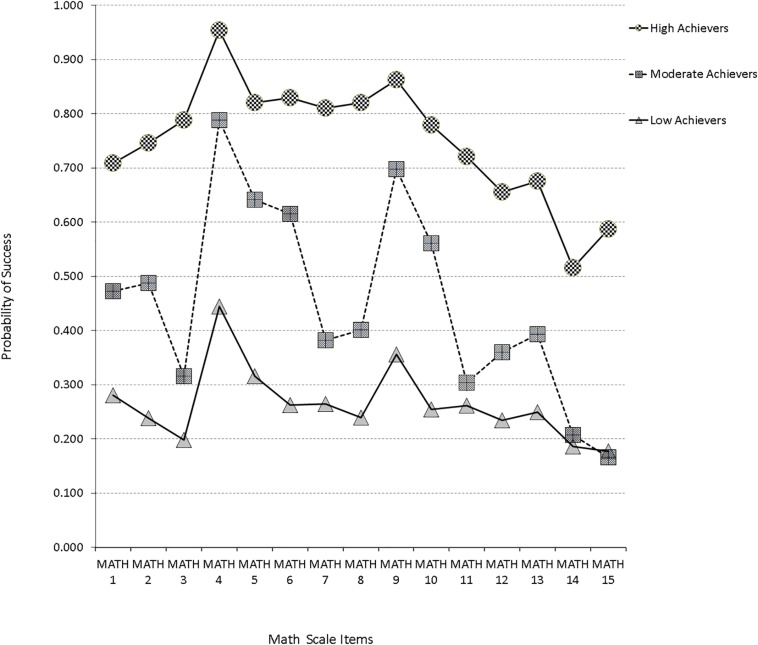
Latent class profile plots for SAAT Mathematics sub-scale based on most commonly used information criteria [e.g., BIC, CAIC, cmP(k)].

Initially, a one-class baseline model was fit to the data as a reference point from which other models would be contrasted (as it is rather uninformative for answering focal hypotheses). When a two-class model was fit to the data, it provided a significantly better model fit, as evidenced by the Vuong-Lo-Mendell-Rubin likelihood ratio test with −2^∗^ the loglikelihood difference been 3764.590, which for 16 parameters, was significantly different from zero (*p* < 0.001). The above finding was further reinforced since all information criteria values of the two-class model were smaller than the corresponding criteria of the one-class model. Similar findings emerged by modeling a third distinct group. Again, the difference likelihood ratio test was significant (−2^∗^LL = 307.106, Δpar = 16, *p* = 0.042). Furthermore, besides the *AIC* which favors larger models, all other information criteria pointed to the superiority of a 3-class model in relation to the 2-class model. When a four-class model was fit to the data, inferential statistics by use of the *LR* difference test favored the 4-class model; however, this effect was not relied upon due to the excessive power linked to the *LRT* test and instead, more value was given to the information criteria. By use of the *BIC*, *CAIC*, and *AWE*, the 3-class model provided the most parsimonious solution with these data. Furthermore, the approximate model probability (cmPk) pointed to the preference of a 3-class solution. Consequently, a 3-group representation provided the best description of different subgroups with these data. The first class (7.5% of the sample) consisted of participants with a high probability of success in all items of the Mathematics scale (high achievers). The second class, which comprised 18.6% of the sample, consisted of participants with moderate probability of success in approximately all scale items (average achievers). Finally, the third class made up of 73.8% of the sample consisted of participants with very low probability of success in the Mathematics scale (low achievers). A graphical representation of the LCA results is presented in Figure 2.

### Testing for DIF

Building upon this best-fitting model, we next examined whether item response patterns within each class were differentiated across the different levels of gender. In other words, we examined whether two participants belonging to the same latent class had the same expected responses on each scale item regardless of their gender (Masyn, 2017). In this case, the latent class indicator (i.e., the item) is considered measurement invariant. In any other case, the item exhibits differential behavior, considered as DIF. As mentioned earlier, this analysis is very important, as if gender is a potential source of DIF, it is important to include direct effects of gender in the latent class regression model, to get unbiased estimates not only for the measurement parameters but also of the structural parameters. Ignoring these effects might lead to misspecification of the latent classes in terms of both the size and the characteristics of each class, which in turn, could lead to misinterpretations of the obtained latent class results.

To examine for possible DIF effects on the Math items within a latent class framework, we adapted the Multiple-Indicator Multiple-Causes (MIMIC) approach (Masyn, 2017), a model which is analogous to the classical MIMIC approach used for DIF detection in factor analysis (MacIntosh and Hashim, 2003; Finch, 2005; Wang et al., 2009; Wang, 2010). The summary of the DIF results across all steps is presented in Table 2.

**TABLE 2:** Model comparisons for Differential Item Functioning (DIF) for gender across classes at a 3-latent class model for mathematics scale.

**Steps**	**Model**	**Model description**	***LL***	***npar***	**Comparison**	***LRTS***	***d.f***	***p-value***
1	M1.0	MIMIC: No DIF	−56743.05	49	M1.0 vs. M1.1	188.58	45	0.001
	M1.1	MIMIC: All DIF	−56648.76	94				
2	M2.0.1	Item1: No DIF	−8349.60	7	M2.0.1 vs. M2.1.1	13.48	3	0.001
	M2.1.1	Item1: N-U DIF	−8342.86	10				
	M2.0.2	Item2: No DIF	−8117.29	7	M2.0.2 vs. M2.1.2	33.56	3	Ns
	M2.1.2	Item2: N-U DIF	−8100.51	10				
	M2.0.3	Item3: No DIF	−7776.14	7	M2.0.3 vs. M2.1.3	2.64	3	Ns
	M2.1.3	Item3: N-U DIF	−7774.82	10				
	M2.0.4	Item4: No DIF	−8208.62	7	M2.0.4 vs. M2.1.4	9.44	3	Ns
	M2.1.4	Item4: N-U DIF	−8203.90	10				
	M2.0.5	Item5: No DIF	−8310.93	7	M2.0.5 vs. M2.1.5	8.84	3	Ns
	M2.1.5	Item5: N-U DIF	−8306.51	10				
	M2.0.6	Item6: No DIF	−8046.94	7	M2.0.6 vs. M2.1.6	0.64	3	Ns
	M2.1.6	Item6: N-U DIF	−8046.62	10				
	M2.0.7	Item7: No DIF	−8189.82	7	M2.0.7 vs. M2.1.7	2.88	3	Ns
	M2.1.7	Item7: N-U DIF	−8188.38	10				
	M2.0.8	Item8: No DIF	−8044.60	7	M2.0.8 vs. M2.1.8	21.60	3	0.001
	M2.1.8	Item8: N-U DIF	−8033.80	10				
	M2.0.9	Item9: No DIF	−8312.74	7	M2.0.9 vs. M2.1.9	19.52	3	0.002
	M2.1.9	Item9: N-U DIF	−8302.98	10				
	M2.0.10	Item10: No DIF	−8127.99	7	M2.0.10 vs. M2.1.10	26.56	3	0.001
	M2.1.10	Item10: N-U DIF	−8114.71	10				
	M2.0.11	Item11: No DIF	−8171.41	7	M2.0.11 vs. M2.1.11	7.38	3	Ns
	M2.1.11	Item11: N-U DIF	−8167.72	10				
	M2.0.12	Item12: No DIF	−8098.77	7	M2.0.12 vs. M2.1.12	2.70	3	Ns
	M2.1.12	Item12: N-U DIF	−8097.42	10				
	M2.0.13	Item13: No DIF	−8171.10	7	M2.0.13 vs. M2.1.13	13.94	3	0.003
	M2.1.13	Item13: N-U DIF	−8164.13	10				
	M2.0.14	Item14: No DIF	−7659.90	7	M2.0.14 vs. M2.1.14	3.56	3	ns
	M2.1.14	Item14: N-U DIF	−7658.12	10				
	M2.0.15	Item15: No DIF	−7490.77	7	M2.0.15 vs. M2.1.15	13.46	3	0.004
	M2.1.15	Item15: N-U DIF	−7484.04	10				
3	M3.0	MIMIC with all N-U DIF items	−56677.56	70	M1.0 vs. M3.0	130.98	21	0.001
					M3.0 vs. M.1.1	57.60	24	0.001
4	M4.1	Item1 (U DIF) all other (N-U DIF)	−56681.62	68	M4.1 vs. M3.0	8.12	2	0.017
	M4.2	Item2 (U DIF) all other (N-U DIF)	−56679.80	68	M4.2 vs. M3.0	4.48	2	ns
	M4.3	Item8 (U DIF) all other (N-U DIF)	−56684.85	68	M4.3 vs. M3.0	14.58	2	0.001
	M4.4.	Item9 (U DIF) all other (N-U DIF)	−56684.02	68	M4.4 vs. M3.0	12.92	2	0.001
	M4.5	Item10 (U DIF) all other (N-U DIF)	−56680.97	68	M4.5 vs. M3.0	6.82	2	0.033
	M4.6	Item13 (U DIF) all other (N-U DIF)	−56679.87	68	M4.6 vs. M3.0	4.62	2	ns
	M4.7.	Item15 (U DIF) all other (N-U DIF)	−56677.72	68	M4.7 vs. M3.0	0.32	2	ns

**LL*, Model maximum log likelihood value; npar, number of free parameters; *LRTS*, likelihood ratio test statistic; d.f., degrees of freedom; MIMIC, Multiple Indicator Multiple Cause; U DIF = uniform DIF; N-U DIF, non-uniform DIF; *p* < 0.001.*

#### Step 1

In this step, we contrasted two models, the Null (M1.0 model), which assumes no-DIF versus an alternative model (M1.1. model), which assumes DIF for all items (termed all-DIF model). The results from the likelihood ratio test statistic (LRTS) suggested a rejection of the Null model M1.0 (*LRTS* = 188.58, *df* = 45, *p* = 0.001), indicating that gender is a source of DIF for at least one of the 15 scale items in at least one of the three latent classes. Thus, we proceeded to Step 2 to decipher item level effects that were responsible for the omnibus DIF finding.

#### Step 2

In this step, we examined each item for non-uniform DIF using two competing models: A Null model, representing No-DIF (M2.0.1) vs. an alternative model (M2.1.1), which represents a non-uniform DIF for a specific item. The results from the likelihood ratio test statistic (LRTS) for all 30 pairwise model comparisons are shown in Step 2 part of Table 2. At the end of this procedure, for eight items (i.e., 3, 4, 5, 6, 7, 11, 12, and 14), the model no-DIF was not statistically significantly worse than the model allowing non-uniform DIF. On the other hand, for 7 Math items (i.e., 1, 2, 8, 9, 10, 13, and 15), the no-DIF model was rejected in favor of the alternative model representing a non-uniform DIF. These findings suggest that the non-uniform DIF items might be functioning differently across gender.

#### Step 3

In this step, we introduced a new model (M3.0), which was based on the findings from Step 2, and aimed to exploit further the non-uniform effects found in Step 2. Particularly, we built a new MIMIC model exclusively from items exhibiting non-uniform DIF. This model was then compared with the MIMIC models M1.0 (no-DIF model) and M1.1 (all-DIF model), respectively. We expected that M3.0 model would be significantly better than model M1.0 (the no-DIF model), but not significantly worse than model M.1.1 (the all-DIF model). When model M3.0 was contrasted to model M1.0 (no-DIF), the later showed improved fit (*LRTS* = 130.98, *df* = 21, *p* = 0.001). When model M3.0 was contrasted to M1.1. (all DIF), significant differences were observed, contrary to expectations (*LRTS* = 57.60, *df* = 24, *p* = 0.001), a finding being likely reflective of excessive power levels observed with over 6,000 participants. This conclusion was further substantiated by examining BIC estimates; Results showed that the BIC value for M3.0 (113,967.05) was lower than the corresponding value for model M1.1 (114,119.26) suggesting that model M3.0 was the preferred model with these data in comparison to M1.1 after accounting for model complexity and sample size. The results from this step are shown in Step 3 part of Table 2. To sum up, all evidence from this step suggests that M3.0 model was the final latent class MIMIC model that summarizes the effects of gender on the latent class solution.

#### Step 4

Last Step in the implementation of the current protocol was to test for uniform DIF (Table 2, step 4). Particularly, we estimated a series of MIMIC models containing only the items exhibiting non-uniform DIF, as described in the previous step (M4.1–M4.7). In these models, we constrained the direct effect from the covariate to each item (one at a time) to be class-invariant, while allowing all other direct effects to vary across classes freely. Then, we compared each model with the M3.0 non-uniform latent class MIMIC model using a difference likelihood ratio test. If the new models (i.e., M4.1 – M 4.7) are not significantly worse than the M3.0 (the non-uniform DIF model), there is evidence for uniform DIF. In contrast, if the new models are statistically worse than the model allowing non-uniform DIF effects, DIF effects are non-uniform. The analysis revealed three uniform DIF items (i.e., 2, 13, and 15) and four non-uniform DIF items (items 1, 8, 9, and 10). Tables 3, 4 present inferential statistics and corresponding effect size indicators for these effects.

**TABLE 3:** Statistics for uniform DIF items across males and females for SAAT’s Mathematics scale.

**Item no**	**Estimate (in logits)**	***95% CIs (UL/LL)***	***S.E.***	***Est./S.E.***	***p-value***	**Effect size**
2	0.307	0.170/0.445	0.070	4.389	0.001	Negligible
13	−0.244	−0.365/−0.123	0.062	−3.952	0.001	Negligible
15	0.271	0.117/426	0.079	3.441	0.001	Negligible

*UL, upper level; LL, low level; S.E, standard error.*

**TABLE 4:** Statistics for non-uniform DIF items across males and females for SAAT’s Mathematics scale.

	**Latent Class 1**			**Latent Class 2**			**Latent Class 3**		
**Item no**	**Estimates (in logits)**	***95% CIs (UL/LL)***	**Effect size**	**Estimates (in logits)**	***95% CIs (UL/LL)***	**Effect size**	**Estimates (in logits)**	***95% CIs (UL/LL)***	**Effect size**
1	−0.075	−0.643/0.493	Negligible	−0.672	−1.076/−0.267	Medium	0.095	−0.100/0.290	Negligible
8	−2.03	−6.779/2.712	Large	−0.335	−0.705/0.034	Negligible	0.425	0.226/0.625	Negligible
9	−0.068	−0.725/0.589	Negligible	−15.09	−15.94/−14.25	Large	−0.176	−0.378/0.027	Negligble
10	−0.266	−0.868/0.335	Negligible	−1.367	−2.242/−0.492	large	−0.073	−0.328/0.181	Negligible

*UL, upper level; LL, low level.*

In terms of uniform DIF, males scored higher than females (positive values designate higher values for males) in items 2 and 15, regardless of their class membership. The opposite occurred in item 13, with females scoring higher than males across all classes. However, when it comes to interpreting the size of DIF in terms of effect size metrics, all three items exhibited negligible uniform DIF (Lin and Lin, 2014). In terms of non-uniform DIF, item 1 exhibited medium level DIF across gender only at latent class 2 (average achievers), with females scoring higher than males. For the remaining classes, the DIF effect was negligible. Item 8 exhibited a large DIF effect only at latent class 1 (high scorers), with females scoring higher than males; in all other classes, the DIF effect was negligible. Items 9 and 10 exhibited large levels DIF in the 2nd class, with females scoring higher than males. In all other classes, the DIF effects were negligible.

## Discussion

The aim of this study was twofold: first, to determine if subgroups of participants completing a standardized admission test for university entrance could be identified based on their performance; the findings from this analysis could help experts, education specialists, and policymakers to identify possible common characteristics shared by participants of each group, and uncover factors/reasons determining their performance; second, to examine whether the observed latent class indicators are invariant across classes in terms of gender. Lately, a standard practice in latent class analysis is to investigate what types of individuals belong to each class by relating classes to covariates (e.g., gender, age, etc.). Thus, this analysis could help experts to identify whether gender is a potential source of DIF for the latent class indicators. Previous findings have shown that ignoring these effects may lead to biased estimated parameters, not only for the measurement but also for the structural model of the latent class analysis (Masyn, 2017; Nylund-Gibson and Choi, 2018). Moreover, if there are DIF items in the latent class model, then the obtained latent classes cannot be used for class comparisons, a practice that is very common in the latent class tradition (e.g., Clark and Muthén, 2009).

To address the first goal, we ran a latent class analysis (LCA). The results from latent class analysis confirmed the heterogeneity of the participants’ performance on this high-stake admission test and revealed a three-class solution. The first class consisted of participants with a high probability of success in SAAT on Math items. It is the smallest class since it is made up of 7.5% of the total sample. The second class, which comprised 18.6% of the sample, consisted of participants with moderate probability of success in all scale items (average achievers). Finally, the third group (largest, comprising 73.8% of the participants) had a low probability of success in the Math items (termed low achievers).

Apart from the above classification of the participants into the three classes, this analysis could provide further information in terms of how specific items performed across the different classes. For example, item 4 was an easy item and had a high probability of success throughout, with even the low ability group having a probability of success greater than 40%. A similar pattern was observed with item 9. On the other hand, item 15 seems to be a difficult item that discriminates high achievers from the other two classes (average and low scorers). However, it does not differentiate efficiently average from low achievers. Similar conclusions can be drawn for item 11. In general, however, the majority of the Math items seem to differentiate participants across classes adequately, suggesting proper class discrimination.

To address the second aim of this study, we examined whether gender is a potential source of measurement non-invariance (DIF). For that, we ran a multiple-indicator multiple-causes model (MIMIC), to examine whether there are direct effects not only from the covariate to the latent class variable but also to the latent class indicators (i.e., scale items). First, we ran an omnibus DIF test, by comparing a model which assumes no-DIF (i.e., gender has an effect on the latent class variable but not direct effects on any of the latent class indicators) versus a model which assumes all-DIF (i.e., gender has an effect on the latent class variable but also has class varying direct effects on all of the latent class indicators). The results showed that the all-DIF model was statistically significantly better than the no-DIF model, indicating that gender is a source of measurement non-invariance (DIF) for at least one of the 15 scale items in at least one of the three latent classes. This is an important finding since it shows that it should be included in the latent class regression model. Unfortunately, the use of covariates in latent class analysis has not yet been established as a standard procedure, although empirical evidence suggests that ignoring the effects of various demographic variables (e.g., gender, age, socio-economic status, income, etc.) can lead to misspecifications of the latent class model classification (Clark and Muthén, 2009; Asparouhov and Muthén, 2014; Masyn, 2017).

Next, we investigated which of the Math items exhibited uniform and non-uniform DIF effects. Uniform DIF is established when the effect of the covariate (i.e., gender) on an item is invariant across classes, while non-uniform DIF is evidenced when the effects of the covariate on an item vary across classes. The results indicated that seven items exhibited significant evidence DIF. From them, three items exhibited uniform DIF (items 2, 13, and 15) and four items non-uniform DIF (items 1, 8, 9, and 10). In terms of the uniform DIF items, males scored higher than females in items 2 and 15, and females scored higher than males in item 13. In all cases, however, the DIF effect size was negligible by the use of effect size conventions. Finally, we examined the nature of non-uniform DIF. First, DIF effects were observed in only one class, with all other classes exhibiting negligible DIF due to gender. Second, in all cases where a medium or strong DIF effect was evidenced, that effect favored females in that they scored higher compared to males.

Findings from this study demonstrated that a latent class analysis could be very useful for providing information about what may be responsible for differential item behavior. Ignoring or avoiding DIF effects in latent class analysis could jeopardize the results of the analysis since it provides biased estimates for the classification process (i.e., the determination of the classes) as well the prediction model (i.e., the relationship between the covariate and the latent class variable). The results from this analysis showed that testing for DIF effects via a MIMIC approach was a necessary procedure to ensure unbiased estimates since there were items exhibiting DIF effects across males and females. Furthermore, it demonstrated that examination of direct effects (measurement non-invariance) from latent class variable predictors (e.g., gender or other demographic variables) to latent class indicators must become a standard procedure in latent class analysis. Potential treatments in the presence of DIF may include (a) purification procedures, (b) merging items with similar content to comprise super items so that DIF effects would dissolve, (c) examining the behavior of the distractors and whether DIF was a function of the item stem or specific distractors, and others.

This study also highlighted the importance of within-person analyses. The results from this study showed that it is not wise to assume that all latent class indicators have the same expected responses across classes and across different levels of a demographic variable such as gender (unfortunately, this is what happens in practice). Thus, the nature of each latent class must be examined by inspecting the manifest characteristics (e.g., demographic information) in each latent class membership. In this study, we showed that response probabilities across latent classes are not the same for all latent class indicators and that individuals within a class could have different response probabilities depending on a specific characteristic (e.g., whether he/she is male or female).

### Limitations and Future Directions

This study has several limitations that need to be pointed out. First, the adapted approach (i.e., MIMIC modeling) is relatively new in the latent class paradigm, with no substantial amount of empirical evidence. Thus, there are some steps in this sequential procedure, for which further evaluation is needed, either with simulation or real-life data. For example, as Masyn (2017) denotes, in Step 2 and perhaps in Step 4, where multiple comparisons are conducted testing for non-uniform and uniform DIF effects for one indicator at a time, it is possible that Type I error inflation is present; thus a sequential *p*-value correction procedure might be necessary such as the Holms sequential Bonferroni procedure or others. In this study, we did not take any action toward that, which might have increased the number of items exhibiting DIF in light of the large sample size. Fortunately, however, the use of the effect size metric in interpreting the magnitude of the DIF has minimized that risk. Second, item content was not available to preserver item non-exposure, and consequently, understanding differences across gender as a function of item content was not currently possible. Third, the class enumeration process was based solely on statistical and not theoretical and content-based criteria for the same reasons just described, and thus, a more thorough and informed solution could not be derived. Fourth, because a large number of participants were associated with excessive power levels when testing for aberrant response patterns and/or the presence of outlying observations, inferential statistics were not implemented in the evaluation process.

Furthermore, outlying cases are likely not influential in the presence of such a large sample size. Consequently, all participants were included in the sample, assuming they are valid members of this large population. Additionally, our use of information criteria, although non-exhaustive, involved several for which little research has been conducted (e.g., AWE) but which were likely more appropriate with large samples. The behavior of the information criteria across conditions and their use for comparative purposes is another unexplored area of inquiry. Last, as the editor noted, the efficacy of the proposed method over competing methodologies would provide insights in relation to the pros and cons of LCA DIF and is therefore a limitation of the present study.

In this study, we examined gender composition as a means of further distinguishing classes. Future work should focus on expanding classification to other demographic variables, such as age, and socio-economic status, etc. Moreover, there is a clear need for incorporating various distal outcomes (e.g., academic performance, academic satisfaction, self-efficacy, etc.) that could be used to examine whether the obtained latent classes display statistically significant mean-level differences and/or whether these processes should be included in the class enumeration process. Such studies could be used to enhance the predictive and discriminant validity of the test. Another interesting line of research, which is related to the further development of the method itself, might be the implementation of the MIMIC modeling in testing DIF effects using continuous variables as covariates (e.g., age) and ordinal or continuous variables as latent class indicators. With that way, the usefulness and utility of this promising approach in measurement theory and practice would be expanded. We see these as some of the many exciting questions awaiting further study.

## Data Availability Statement

The datasets generated for this study are available on request to the corresponding author.

## Ethics Statement

The studies involving human participants were reviewed and approved by The National Center for Assessment in Higher Education (Qiyas) Ethics Committee (www.qiyas.sa). The patients/participants provided their written informed consent to participate in this study.

## Author Contributions

IT designed the research project, performed the statistical analysis, and completed the original version of the manuscript. GS contributed to the development of the article, the write-up of the methods section, polished, revised and approved the final version of the manuscript. HA collected the data, drafted parts of the sections “Introduction” and “Materials and Methods,” proofread the entire manuscript, and approved all parts of the written product.

## Conflict of Interest

The authors declare that the research was conducted in the absence of any commercial or financial relationships that could be construed as a potential conflict of interest.

## Footnotes

1 www.qiyas.sa
