# Supplementary material for: Measurement Invariance and Differential Item Functioning Across Gender Within a Latent Class Analysis Framework: Evidence From a High-Stakes Test for University Admission in Saudi Arabia
Source: Front Psychol. 2020 Apr 3;11:622. doi: 10.3389/fpsyg.2020.00622 (PMC7147614; doi:10.3389/fpsyg.2020.00622)
Supplement: Supplementary file 1 [file Table_1.DOCX]

**Mplus Syntax for Stepwise MIMIC Model DIF Detection**

**STEP 0 (Model M.0)**

TITLE: Mathematics Domain - Stepwise MIMIC Model DIF Detection (M0)

DATA: file is MATH data.dat;

VARIABLE:

NAMES ARE id gender math1 math2 math3 math4 math5 math6 math7 math8 math9 math10

math11 math12 math13 math14 math15;

MISSING ARE ALL (99);

IDVARIABLE = id;

Auxiliary = gender;

USEVARIABLES ARE math1 math2 math3 math4 math5 math6 math7 math8 math9 math10

math11 math12 math13 math14 math15;

CATEGORICAL ARE math1 math2 math3 math4 math5 math6 math7 math8 math9 math10

math11 math12 math13 math14 math15;

CLASSES = c(3);

ANALYSIS:

type=mixture;

STARTS=0;

processors = 7;

MODEL:

%OVERALL%

[ c#1*-2.18216 ];

[ c#2*-1.12108 ];

%C#1%

[ math1$1*-0.88963 ];

[ math2$1*-1.07618 ];

[ math3$1*-1.31355 ];

[ math4$1*-3.03987 ];

[ math5$1*-1.51336 ];

[ math6$1*-1.57537 ];

[ math7$1*-1.44816 ];

[ math8$1*-1.51554 ];

[ math9$1*-1.82938 ];

[ math10$1*-1.25932 ];

[ math11$1*-0.94991 ];

[ math12$1*-0.64262 ];

[ math13$1*-0.73615 ];

[ math14$1*-0.06561 ];

[ math15$1*-0.35321 ];

%C#2%

[ math1$1*0.10739 ];

[ math2$1*0.04643 ];

[ math3$1*0.77142 ];

[ math4$1*-1.31459 ];

[ math5$1*-0.57856 ];

[ math6$1*-0.46744 ];

[ math7$1*0.48280 ];

[ math8$1*0.40047 ];

[ math9$1*-0.83943 ];

[ math10$1*-0.24644 ];

[ math11$1*0.82774 ];

[ math12$1*0.57662 ];

[ math13$1*0.43315 ];

[ math14$1*1.34414 ];

[ math15$1*1.61516 ];

%C#3%

[ math1$1*0.93991 ];

[ math2$1*1.16096 ];

[ math3$1*1.39745 ];

[ math4$1*0.22304 ];

[ math5$1*0.77431 ];

[ math6$1*1.03014 ];

[ math7$1*1.02162 ];

[ math8$1*1.15925 ];

[ math9$1*0.59237 ];

[ math10$1*1.07168 ];

[ math11$1*1.03407 ];

[ math12$1*1.18379 ];

[ math13$1*1.10236 ];

[ math14$1*1.47642 ];

[ math15$1*1.53790 ];

OUTPUT:

TECH1 TECH8;

PLOT: type=plot3;

series = math1 (1) math2 (2) math3 (3) math4 (4) math5 (5) math6 (6) math7 (7) math8 (8)

math9 (9) math10 (10) math11 (11) math12 (12) math13 (13) math14 (14) math15 (15);

! how the variables are presented in the X axis

! (*) separate them by a space

SAVEDATA:

file = MATH data_savedata.txt;

save = cprob;

missflag = 9999;

format = free;

**STEP 1 (Model M1.0)**

TITLE: Mathematics Domain - Stepwise MIMIC Model DIF Detection (M1.0)

DATA: file is MATH data.dat;

VARIABLE:

NAMES ARE id gender math1 math2 math3 math4 math5 math6 math7 math8 math9 math10

math11 math12 math13 math14 math15;

MISSING ARE ALL (99);

IDVARIABLE = id;

USEVARIABLES ARE gender math1 math2 math3 math4 math5 math6 math7 math8 math9 math10 math11 math12 math13 math14 math15;

CATEGORICAL ARE math1 math2 math3 math4 math5 math6 math7 math8 math9 math10

math11 math12 math13 math14 math15;

CLASSES = c(3);

ANALYSIS:

type=mixture;

STARTS=0;

processors = 7;

MODEL:

%OVERALL%

[ c#1*-2.18216 ];

[ c#2*-1.12108 ];

c on gender;

%C#1%

[ math1$1*-0.88963 ];

[ math2$1*-1.07618 ];

[ math3$1*-1.31355 ];

[ math4$1*-3.03987 ];

[ math5$1*-1.51336 ];

[ math6$1*-1.57537 ];

[ math7$1*-1.44816 ];

[ math8$1*-1.51554 ];

[ math9$1*-1.82938 ];

[ math10$1*-1.25932 ];

[ math11$1*-0.94991 ];

[ math12$1*-0.64262 ];

[ math13$1*-0.73615 ];

[ math14$1*-0.06561 ];

[ math15$1*-0.35321 ];

%C#2%

[ math1$1*0.10739 ];

[ math2$1*0.04643 ];

[ math3$1*0.77142 ];

[ math4$1*-1.31459 ];

[ math5$1*-0.57856 ];

[ math6$1*-0.46744 ];

[ math7$1*0.48280 ];

[ math8$1*0.40047 ];

[ math9$1*-0.83943 ];

[ math10$1*-0.24644 ];

[ math11$1*0.82774 ];

[ math12$1*0.57662 ];

[ math13$1*0.43315 ];

[ math14$1*1.34414 ];

[ math15$1*1.61516 ];

%C#3%

[ math1$1*0.93991 ];

[ math2$1*1.16096 ];

[ math3$1*1.39745 ];

[ math4$1*0.22304 ];

[ math5$1*0.77431 ];

[ math6$1*1.03014 ];

[ math7$1*1.02162 ];

[ math8$1*1.15925 ];

[ math9$1*0.59237 ];

[ math10$1*1.07168 ];

[ math11$1*1.03407 ];

[ math12$1*1.18379 ];

[ math13$1*1.10236 ];

[ math14$1*1.47642 ];

[ math15$1*1.53790 ];

**STEP 1 (Model M1.1)**

TITLE: Mathematics Domain - Stepwise MIMIC Model DIF Detection (M1.1)

DATA: file is MATH data.dat;

VARIABLE:

NAMES ARE id gender math1 math2 math3 math4 math5 math6 math7 math8 math9 math10

math11 math12 math13 math14 math15;

MISSING ARE ALL (99);

IDVARIABLE = id;

USEVARIABLES ARE gender math1 math2 math3 math4 math5 math6 math7 math8 math9 math10 math11 math12 math13 math14 math15;

CATEGORICAL ARE math1 math2 math3 math4 math5 math6 math7 math8 math9 math10

math11 math12 math13 math14 math15;

CLASSES = c(3);

ANALYSIS:

type=mixture;

STARTS=0;

processors = 7;

MODEL:

%OVERALL%

[ c#1*-2.18216 ];

[ c#2*-1.12108 ];

c on gender;

math1 math2 math3 math4 math5 math6 math7 math8 math9 math10 math11 math12 math13 math14 math15 on gender;

%C#1%

[ math1$1*-0.88963 ];

[ math2$1*-1.07618 ];

[ math3$1*-1.31355 ];

[ math4$1*-3.03987 ];

[ math5$1*-1.51336 ];

[ math6$1*-1.57537 ];

[ math7$1*-1.44816 ];

[ math8$1*-1.51554 ];

[ math9$1*-1.82938 ];

[ math10$1*-1.25932 ];

[ math11$1*-0.94991 ];

[ math12$1*-0.64262 ];

[ math13$1*-0.73615 ];

[ math14$1*-0.06561 ];

[ math15$1*-0.35321 ];

math1 math2 math3 math4 math5 math6 math7 math8 math9 math10 math11 math12 math13 math14 math15 on gender;

%C#2%

[ math1$1*0.10739 ];

[ math2$1*0.04643 ];

[ math3$1*0.77142 ];

[ math4$1*-1.31459 ];

[ math5$1*-0.57856 ];

[ math6$1*-0.46744 ];

[ math7$1*0.48280 ];

[ math8$1*0.40047 ];

[ math9$1*-0.83943 ];

[ math10$1*-0.24644 ];

[ math11$1*0.82774 ];

[ math12$1*0.57662 ];

[ math13$1*0.43315 ];

[ math14$1*1.34414 ];

[ math15$1*1.61516 ];

math1 math2 math3 math4 math5 math6 math7 math8 math9 math10 math11 math12 math13 math14 math15 on gender;

%C#3%

[ math1$1*0.93991 ];

[ math2$1*1.16096 ];

[ math3$1*1.39745 ];

[ math4$1*0.22304 ];

[ math5$1*0.77431 ];

[ math6$1*1.03014 ];

[ math7$1*1.02162 ];

[ math8$1*1.15925 ];

[ math9$1*0.59237 ];

[ math10$1*1.07168 ];

[ math11$1*1.03407 ];

[ math12$1*1.18379 ];

[ math13$1*1.10236 ];

[ math14$1*1.47642 ];

[ math15$1*1.53790 ];

math1 math2 math3 math4 math5 math6 math7 math8 math9 math10 math11 math12 math13 math14 math15 on gender;

**STEP 2 (Model M2.0)**

! Same as Step 0, but using SAVEDATA option instead of actual data file

TITLE: Mathematics Domain - Stepwise MIMIC Model DIF Detection (M2.0)

DATA: file is data_savedata.dat;

VARIABLE:

NAMES ARE id gender math1 math2 math3 math4 math5 math6 math7 math8 math9 math10

math11 math12 math13 math14 math15;

MISSING ARE ALL (99);

IDVARIABLE = id;

Auxiliary = gender;

USEVARIABLES ARE math1 math2 math3 math4 math5 math6 math7 math8 math9 math10

math11 math12 math13 math14 math15;

CATEGORICAL ARE math1 math2 math3 math4 math5 math6 math7 math8 math9 math10

math11 math12 math13 math14 math15;

CLASSES = c(3);

ANALYSIS:

type=mixture;

STARTS=0;

processors = 7;

MODEL:

%OVERALL%

[ c#1*-2.18216 ];

[ c#2*-1.12108 ];

%C#1%

[ math1$1*-0.88963 ];

[ math2$1*-1.07618 ];

[ math3$1*-1.31355 ];

[ math4$1*-3.03987 ];

[ math5$1*-1.51336 ];

[ math6$1*-1.57537 ];

[ math7$1*-1.44816 ];

[ math8$1*-1.51554 ];

[ math9$1*-1.82938 ];

[ math10$1*-1.25932 ];

[ math11$1*-0.94991 ];

[ math12$1*-0.64262 ];

[ math13$1*-0.73615 ];

[ math14$1*-0.06561 ];

[ math15$1*-0.35321 ];

%C#2%

[ math1$1*0.10739 ];

[ math2$1*0.04643 ];

[ math3$1*0.77142 ];

[ math4$1*-1.31459 ];

[ math5$1*-0.57856 ];

[ math6$1*-0.46744 ];

[ math7$1*0.48280 ];

[ math8$1*0.40047 ];

[ math9$1*-0.83943 ];

[ math10$1*-0.24644 ];

[ math11$1*0.82774 ];

[ math12$1*0.57662 ];

[ math13$1*0.43315 ];

[ math14$1*1.34414 ];

[ math15$1*1.61516 ];

%C#3%

[ math1$1*0.93991 ];

[ math2$1*1.16096 ];

[ math3$1*1.39745 ];

[ math4$1*0.22304 ];

[ math5$1*0.77431 ];

[ math6$1*1.03014 ];

[ math7$1*1.02162 ];

[ math8$1*1.15925 ];

[ math9$1*0.59237 ];

[ math10$1*1.07168 ];

[ math11$1*1.03407 ];

[ math12$1*1.18379 ];

[ math13$1*1.10236 ];

[ math14$1*1.47642 ];

[ math15$1*1.53790 ];

OUTPUT:

TECH1 TECH8;

PLOT: type=plot3;

series = math1 (1) math2 (2) math3 (3) math4 (4) math5 (5) math6 (6) math7 (7) math8 (8)

math9 (9) math10 (10) math11 (11) math12 (12) math13 (13) math14 (14) math15 (15);

! how the variables are presented in the X axis

! (*) separate them by a space

SAVEDATA:

file = MATH data_savedata.txt;

save = cprob;

missflag = 9999;

format = free;

**STEP 2.1 (Model M2.0.1)**

TITLE: Mathematics Domain - Stepwise MIMIC Model DIF Detection (M2.0.1)

DATA: file is MATH data_savedata.dat;

VARIABLE:

NAMES ARE math1 math2 math3 math4 math5 math6 math7 math8 math9 math10

math11 math12 math13 math14 math15 gender cprob1 cprob2 cprob3 cmod id;

MISSING ARE ALL (9999);

IDVARIABLE = id;

USEVARIABLES ARE math1 cmod gender;

CATEGORICAL ARE math1;

NOMINAL are cmod;

CLASSES = c(3);

ANALYSIS:

type=mixture;

STARTS=0;

processors = 7;

MODEL:

%OVERALL%

[ c#1*-2.18216 ];

[ c#2*-1.12108 ];

c on gender;

%C#1%

[cmod#1@4.401 cmod#2@2.939];

%C#2%

[cmod#1@-2.046 cmod#2@0.392];

%C#3%

[cmod#1@-6.534 cmod#2@-2.709];

! Note: This step is repeated for each item separately.

**STEP 2.1 (Model M2.1.1)**

TITLE: Mathematics Domain - Stepwise MIMIC Model DIF Detection (M2.1.1)

DATA: file is MATH data_savedata.dat;

VARIABLE:

NAMES ARE math1 math2 math3 math4 math5 math6 math7 math8 math9 math10

math11 math12 math13 math14 math15 gender cprob1 cprob2 cprob3 cmod id;

MISSING ARE ALL (9999);

IDVARIABLE = id;

USEVARIABLES ARE math1 cmod gender;

CATEGORICAL ARE math1;

NOMINAL are cmod;

CLASSES = c(3);

ANALYSIS:

type=mixture;

STARTS=0;

processors = 7;

MODEL:

%OVERALL%

[ c#1*-2.18216 ];

[ c#2*-1.12108 ];

c on gender;

math1 on gender;

%C#1%

[cmod#1@4.401 cmod#2@2.939];

math1 on gender;

%C#2%

[cmod#1@-2.046 cmod#2@0.392];

math1 on gender;

%C#3%

[cmod#1@-6.534 cmod#2@-2.709];

math1 on gender;

OUTPUT: CINTERVAL;

! Note: This step is repeated for each item separately.

**STEP 3 (Model M3.0)**

TITLE: Mathematics Domain - Stepwise MIMIC Model DIF Detection (M3.0)

DATA: file is MATH data.dat;

VARIABLE:

NAMES ARE id gender math1 math2 math3 math4 math5 math6 math7 math8 math9 math10

math11 math12 math13 math14 math15;

MISSING ARE ALL (99);

IDVARIABLE = id;

USEVARIABLES ARE gender math1 math2 math3 math4 math5 math6 math7 math8 math9 math10 math11 math12 math13 math14 math15;

CATEGORICAL ARE math1 math2 math3 math4 math5 math6 math7 math8 math9 math10

math11 math12 math13 math14 math15;

CLASSES = c(3);

ANALYSIS:

type=mixture;

STARTS=0;

processors = 7;

MODEL:

%OVERALL%

[ c#1*-2.18216 ];

[ c#2*-1.12108 ];

c on gender;

math1 math2 math8 math9 math10 math13 math15 on gender;

%C#1%

[ math1$1*-0.88963 ];

[ math2$1*-1.07618 ];

[ math3$1*-1.31355 ];

[ math4$1*-3.03987 ];

[ math5$1*-1.51336 ];

[ math6$1*-1.57537 ];

[ math7$1*-1.44816 ];

[ math8$1*-1.51554 ];

[ math9$1*-1.82938 ];

[ math10$1*-1.25932 ];

[ math11$1*-0.94991 ];

[ math12$1*-0.64262 ];

[ math13$1*-0.73615 ];

[ math14$1*-0.06561 ];

[ math15$1*-0.35321 ];

math1 math2 math8 math9 math10 math13 math15 on gender;

%C#2%

[ math1$1*0.10739 ];

[ math2$1*0.04643 ];

[ math3$1*0.77142 ];

[ math4$1*-1.31459 ];

[ math5$1*-0.57856 ];

[ math6$1*-0.46744 ];

[ math7$1*0.48280 ];

[ math8$1*0.40047 ];

[ math9$1*-0.83943 ];

[ math10$1*-0.24644 ];

[ math11$1*0.82774 ];

[ math12$1*0.57662 ];

[ math13$1*0.43315 ];

[ math14$1*1.34414 ];

[ math15$1*1.61516 ];

math1 math2 math8 math9 math10 math13 math15 on gender;

%C#3%

[ math1$1*0.93991 ];

[ math2$1*1.16096 ];

[ math3$1*1.39745 ];

[ math4$1*0.22304 ];

[ math5$1*0.77431 ];

[ math6$1*1.03014 ];

[ math7$1*1.02162 ];

[ math8$1*1.15925 ];

[ math9$1*0.59237 ];

[ math10$1*1.07168 ];

[ math11$1*1.03407 ];

[ math12$1*1.18379 ];

[ math13$1*1.10236 ];

[ math14$1*1.47642 ];

[ math15$1*1.53790 ];

math1 math2 math8 math9 math10 math13 math15 on gender;

**STEP 4 (Model M4.1)**

TITLE: Mathematics Domain - Stepwise MIMIC Model DIF Detection (M4.1)

DATA: file is MATH data.dat;

VARIABLE:

NAMES ARE id gender math1 math2 math3 math4 math5 math6 math7 math8 math9 math10

math11 math12 math13 math14 math15;

MISSING ARE ALL (99);

IDVARIABLE = id;

USEVARIABLES ARE gender math1 math2 math3 math4 math5 math6 math7 math8 math9 math10 math11 math12 math13 math14 math15;

CATEGORICAL math1 math2 math3 math4 math5 math6 math7 math8 math9 math10

math11 math12 math13 math14 math15;

CLASSES = c(3);

ANALYSIS:

type=mixture;

STARTS=0;

processors = 7;

MODEL:

%OVERALL%

[ c#1*-2.18216 ];

[ c#2*-1.12108 ];

c on gender;

math1 math2 math8 math9 math10 math13 math15 on gender;

%C#1%

[ math1$1*-0.88963 ];

[ math2$1*-1.07618 ];

[ math3$1*-1.31355 ];

[ math4$1*-3.03987 ];

[ math5$1*-1.51336 ];

[ math6$1*-1.57537 ];

[ math7$1*-1.44816 ];

[ math8$1*-1.51554 ];

[ math9$1*-1.82938 ];

[ math10$1*-1.25932 ];

[ math11$1*-0.94991 ];

[ math12$1*-0.64262 ];

[ math13$1*-0.73615 ];

[ math14$1*-0.06561 ];

[ math15$1*-0.35321 ];

math2 math8 math9 math10 math13 math15 on gender;

%C#2%

[ math1$1*0.10739 ];

[ math2$1*0.04643 ];

[ math3$1*0.77142 ];

[ math4$1*-1.31459 ];

[ math5$1*-0.57856 ];

[ math6$1*-0.46744 ];

[ math7$1*0.48280 ];

[ math8$1*0.40047 ];

[ math9$1*-0.83943 ];

[ math10$1*-0.24644 ];

[ math11$1*0.82774 ];

[ math12$1*0.57662 ];

[ math13$1*0.43315 ];

[ math14$1*1.34414 ];

[ math15$1*1.61516 ];

math2 math8 math9 math10 math13 math15 on gender;

%C#3%

[ math1$1*0.93991 ];

[ math2$1*1.16096 ];

[ math3$1*1.39745 ];

[ math4$1*0.22304 ];

[ math5$1*0.77431 ];

[ math6$1*1.03014 ];

[ math7$1*1.02162 ];

[ math8$1*1.15925 ];

[ math9$1*0.59237 ];

[ math10$1*1.07168 ];

[ math11$1*1.03407 ];

[ math12$1*1.18379 ];

[ math13$1*1.10236 ];

[ math14$1*1.47642 ];

[ math15$1*1.53790 ];

math2 math8 math9 math10 math13 math15 on gender;

! Note: This step is repeated only for items exhibiting non-uniform DIF.
